# Early Treatment Response Monitoring Using 2-Deoxy-2-[^**18**^F]fluoro-D-glucose Positron Emission Tomography Imaging during Fractionated Radiotherapy of Head Neck Cancer Xenografts

**DOI:** 10.1155/2014/598052

**Published:** 2014-04-28

**Authors:** Jiayi Huang, John L. Chunta, Mitual Amin, David Y. Lee, Inga S. Grills, Ching-Yee Oliver Wong, Brian Marples, Di Yan, George D. Wilson

**Affiliations:** ^1^Department of Radiation Oncology, William Beaumont Hospital, 3811 West Thirteen Mile Road, 105-RI, Royal Oak, MI 48073, USA; ^2^Department of Pathology, William Beaumont Hospital, Royal Oak, MI 48073, USA; ^3^Department of Nuclear Medicine, William Beaumont Hospital, Royal Oak, MI 48073, USA

## Abstract

*Background.* To determine the optimal timing and analytic method of 2-deoxy-2-[^18^F]fluoro-D-glucose positron emission tomography (PET) imaging during fractionated radiotherapy (RT) to predict tumor control. *Methods.* Ten head neck squamous cell carcinoma xenografts derived from the UT-14-SCC cell line were irradiated with 50 Gy at 2 Gy per day over 5 weeks. Dynamic PET scans were acquired over 70 minutes at baseline (week 0) and weekly for seven weeks. PET data were analyzed using standard uptake value (SUV), retention index (RI), sensitivity factor (SF), and kinetic index (Ki). *Results.* Four xenografts had local failure (LF) and 6 had local control. Eighty scans from week 0 to week 7 were analyzed. RI and SF after 10 Gy appeared to be the optimal predictors for LF. In contrast, SUV and Ki during RT were not significant predictors for LF. *Conclusion.* RI and SF of PET obtained after the first week of fractionated RT were the optimal methods and timing to predict tumor control.

## 1. Introduction

Head neck squamous cell carcinoma (HNSCC) has an annual incidence of more than 600,000 cases worldwide [1]. When organ preservation is desired or when surgery is not an option, radiation therapy (RT) alone or definitive chemoradiotherapy is typically the treatment of choice for early stage or advanced stage HNSCC, respectively [2]. Locoregional recurrence can occur in 10–50% of cases after RT or chemoradiotherapy depending on the tumor site and stage. Recently, the emergence of intensity-modulated RT [3] and molecular-targeted therapy [4] has provided hope for improving cure rate. However, to maximize the potential of novel therapy, noninvasive imaging methods that can accurately monitor early treatment response would be crucial to promote individualized therapy.

Positron emission tomography (PET) using 2-deoxy-2-[^18^F]fluoro-D-glucose (FDG) is an attractive imaging method given its ability to provide metabolic and possible biological information [5]. However, imaging with FDG-PET during RT can be confounded by many factors such as inflammatory or vasculature changes [6, 7]. Clinical studies of HNSCC patients have previously tested the utility of early PET during RT to predict clinical outcome and have yielded mixed results [8–11]. Most studies have relied on standard uptake value (SUV) from static PET scans [9–11] or have used a single time point chosen empirically for scanning during RT [8–10]. Understandably, serial dynamic PET scans during seven weeks of fractionated RT would be very expensive and logistically difficult in clinical settings. To our knowledge, the optimal time point for PET scanning during RT and the best PET parameter to evaluate for early treatment response remain unclear and have not been exhaustively studied before.

We have previously reported a translational study in which we correlated FDG-PET of HNSCC xenografts directly with histology at different time points after a single dose of subcurative RT [12]. In that study, a variety of PET parameters in addition to SUV were analyzed using dynamic PET data collected over two hours. The results showed that some of the more complex parameters such as kinetic index (Ki), sensitivity factor (SF), and retention index (RI) appeared to correlate with early radiation-induced necrosis or cellular change on histology [12]. To build upon the promising findings, we designed a pilot translational study to analyze weekly changes of these more experimental parameters of FDG-PET during five weeks of fractionated RT and to correlate with the ultimate tumor response. The goal of the pilot study was to determine the optimal scanning time point and the most promising PET parameter to assess for early treatment response of HNSCC in future studies or clinical trials.

## 2. Materials and Methods

### 2.1. Experimental Design

**Figure 1:**
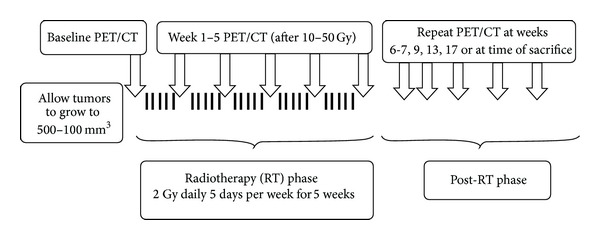
Experimental schema.

Ten xenografts were established on the flanks of athymic female *nu*/*nu* mice using UT-SCC-14 cell line (a low passage head neck cancer cell line) as previously described [12]. The UT-SCC-14 cells have a reported TCD_50_ (the dose required to produce 50% tumor control locally) of 52 gray (Gy) using fractionated irradiation over 6 weeks [13]. The schema of the study is illustrated in Figure 1. Tumors were allowed to grow to approximately 500−1000 mm^3^ (approximately between 5 and 6 mm diameter) before radiation therapy (RT) due to logistics and to mimic the tumor size heterogeneity in the clinical setting. All ten mice were then irradiated with 5 weeks of conventionally fractionated RT; FDG-PET and computed tomography (CT) scans were performed on the mice before the start of RT, weekly during RT, and at selected time points after RT as shown in Figure 1. Each PET scan was performed at least 36 hours after the last administration of RT, which was designed to minimize acute rise of metabolic activity immediately after RT [6, 14]. The primary endpoint of the study was local control of xenografts. The study was initially designed to observe tumor regrowth for a period of 3 months after RT but subsequently extended to 6 months to detect any late tumor recurrence. Tumors were excised prior to 6 months if they were causing symptoms requiring sacrifice or if mice died of other causes. All animal experiments were conducted with the approval and oversight of the Institutional Animal Care and Use Committee.

### 2.2. Radiotherapy

A dose of 50 Gy in 2 Gy fractions, 5 days per week over 5 weeks, was chosen to be biologically equivalent to the published TCD_50_ data [13]. RT was applied using the Faxitron Cabinet X-ray System, Model 43855F (Faxitron X-Ray, Wheeling, IL), at a dose rate of 0.69 Gy/min. The tube voltage was 160 KVp, the current was 4 mA, and filtration was 0.5 mM Al plus 0.5 mM Cu (HVL = 0.77 mm CU). For irradiation, unanaesthetized animals were immobilized in custom-designed jigs with only the tumor and hind flank exposed to the radiation beam.

### 2.3. Small-Animal PET Imaging and Analysis

FDG-PET and CT scans were performed with ^18^F-FDG and using FLEX* Triumph* trimodality microPET/SPECT/CT system (GE Health/Gamma Medica-Ideas, Waukesha, WI). The PET data were reconstructed using a two-dimensional MLEM algorithm resulting in a voxel size of 0.5 mm × 0.5 mm × 1.2 mm (*x*, *y*, *z*) and an actual spatial resolution of approximately 1 mm. The technical details of FDG-PET acquisition and analysis have been described previously [12]. Briefly, the animals under fasting condition were anesthetized with 1-2% isoflurane (balance, 100% O_2_) and were positioned in the center of PET ring field of view with monitoring of body temperature and respiratory rate. PET imaging was initiated with a 5 s delay before injecting 22.9 MBq (±10%) of ^18^F-FDG in 0.2 mL (0.4 mL/min) with an automated infusion pump (Model #70-2204, Harvard Apparatus, Holliston, MA). Based on our previous experience, FDG-PET data were dynamically acquired for 70 minutes after injection. Dynamic FDG-PET data were analyzed using PMOD (PMOD Technologies, Zurich, Switzerland) to generate tumor time activity curves with the aid of CT-derived volumes using VIVID (GE Healthcare/Gamma Medica-Ideas, Waukesha, WI). The following PET parameters were analyzed: SUV, RI, SF, and Ki. All the PET parameters were analyzed using the maximum top 5% and 10% voxel activity within the tumor. The maximum value of SUV (SUV_max⁡_) was calculated as the tumor tissue FDG activity at 60 min after injection divided by the injected dose per body weight of the mouse [15]. RI was defined as (SUV_max⁡_ at 70 min⁡− SUV_max⁡_ at 12 min)/(SUV_max⁡_ at 12 min) × 100% [16]. SF was calculated using a regression of log-transformed data of the change of activity from 12 to 70 min [17]. SF is mathematically related to RI but is calculated using the dynamic data instead of two static data points [17]. Ki was calculated using Patlak analysis, using the dynamic data from 12 to 70 min with the input function derived from the aorta [18].

### 2.4. Local Control and Local Failure Definition

The UT-14-SCC xenografts grow with a volume-doubling time of 4.8 ± 0.7 days. The primary endpoint of the study was local control of xenografts. Local control was defined if the tumor had disappeared after RT (confirmed by dissection) or if the residual tumor had no viable tumor cells upon histological examination. Local failure was defined if the tumor had regrown after RT or if the residual tumor contained viable tumor cells. All tumors at the completion of the study were processed through xylene/alcohols and then embedded in paraffin. Paraffin sections (5 *μ*m) were cut and stained with hematoxylin and eosin (H&E) for histological confirmation. All H&E slides were reviewed by a board-certified pathologist.

### 2.5. Statistical Analysis

Predictive accuracy of FDG-PET was evaluated using the receiver operating characteristic (ROC) analysis with the area under the ROC curve (AUC) as an index of accuracy. The Wilcoxon test was used to compare tumor volumes or FDG-PET parameters between local control and local failure groups. Rates of local control were estimated using the Kaplan-Meier analysis and compared using the log-rank test. Time to local failure was calculated from the date of RT completion. Statistical significance was considered when the *P* value was less than or equal to 0.05. Statistical analyses were performed with SPSS version 18.0 (SPSS Inc., Chicago, IL), and all levels of significance were two-sided.

## 3. Results

Of the 10 mice with xenografts, 4 had local failures after RT and 6 were locally controlled. Of the 4 local failures, 2 tumors regrew (8 and 19 weeks after RT, resp.) and two other tumors were excised before tumor regrowth due to clinical symptoms (9 and 15 weeks after RT, resp.). All 4 tumors were confirmed to have viable tumor cells present on histological examination. Of the 6 locally controlled tumors, 5 tumors completely disappeared (median time to complete clinical response: 7 weeks after RT; range: 3–19 weeks); one persisted as a fibrotic nodule at the end of the study (23 weeks after RT) but contained no viable tumor upon histological evaluation. A total of 104 PET/CT scans were performed. All 10 mice successfully underwent weekly PET/CT imaging from week 0 (baseline) through week 7 (2 weeks after the completion of RT). Since the primary goal was to investigate PET for predicting early radiation response, the analysis focused on those 80 PET scans from week 0 to week 7.

**Figure 2:**
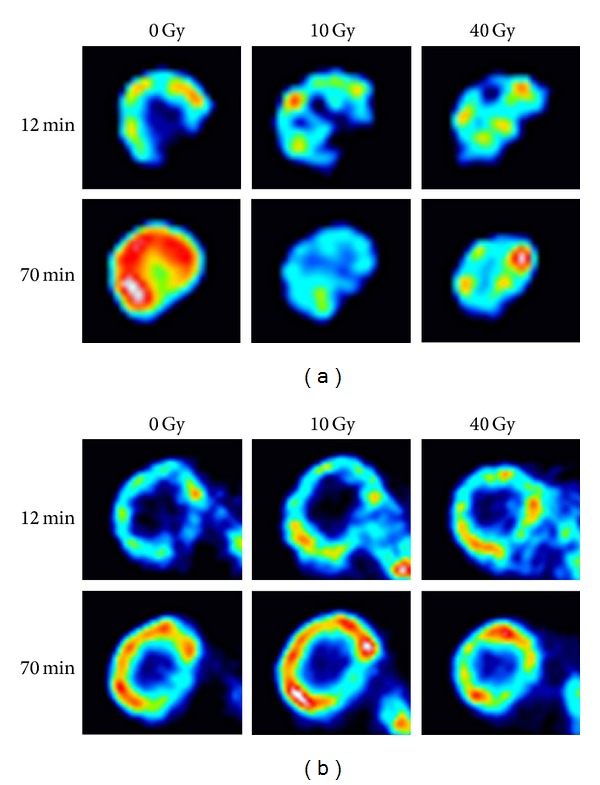
Comparison of FDG-PET changes over time between locally controlled versus uncontrolled tumors. The images represent raw tumor activities with window and level automatically set to reflect all activity values for the images at 12 minutes. The same settings are then applied to each corresponding image at 70 minutes from the same dynamic PET scan. (a) A locally controlled tumor. (b) A locally uncontrolled tumor. PET images prior to radiotherapy (0 Gy), at week 1 (10 Gy) and at week 4, (40 Gy) are shown as tumor activity at 12 min and 70 min (after injection).

**Figure 3:**
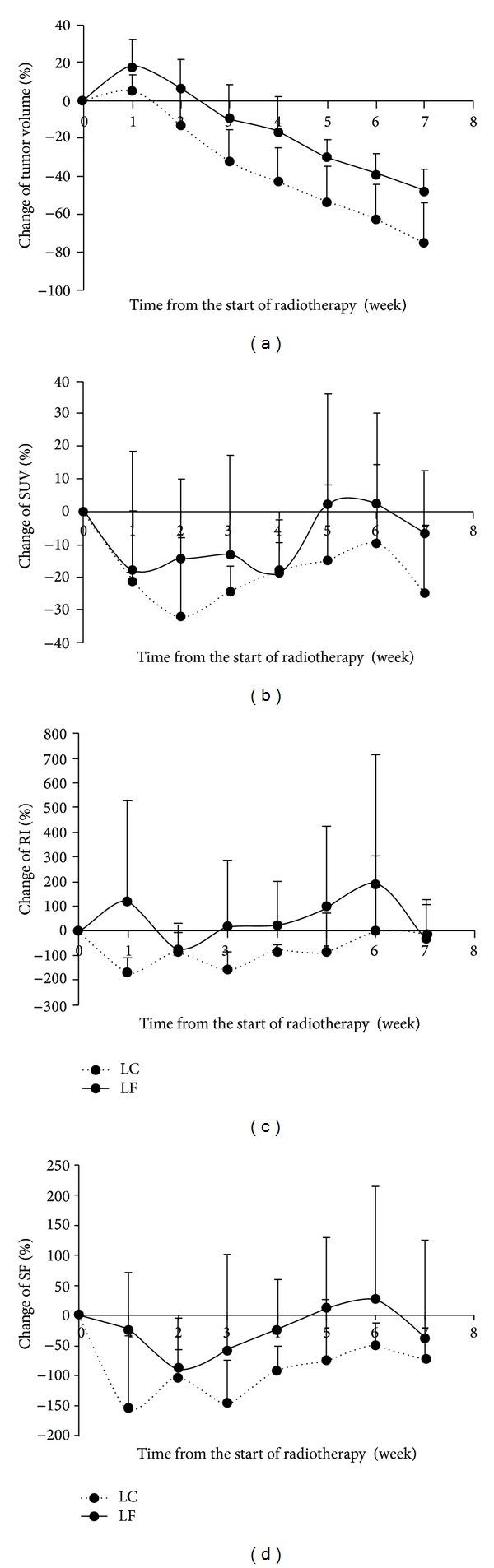
Percent changes of tumor volume and selected PET parameters during and immediately after radiotherapy. (a) Tumor volume. (b) Standard uptake value (SUV). (c) Retention index (RI). (d) Sensitivity factor (SF). A solid line represents tumors with local failure (LF); dashed line represents tumors with local control (LC). Points are shown as means with error bars indicating SE and only positive SE are shown for clarity.

Figure 2 contrasts the dynamic changes of tumor activity of FDG-PET between a controlled tumor versus an uncontrolled one at different time points during RT. After 10 Gy, the activity of the controlled tumor decreases between 12 and 70 min (Figure 2(a)), whereas the activity of the uncontrolled tumor increases over time (Figure 2(b)). Similar but less dramatic changes are noted after 40 Gy. The PET images are indicative of central necrosis which confirms a key finding from our previous FDG-PET imaging study where we related PET parameters to histological staining [12].  Table 1 demonstrates that neither pretreatment tumor volumes nor PET parameters are significantly different between the local failure and local control groups.  Figure 3 depicts the percent changes of tumor volume, SUV, RI, and SF during the first 7 weeks. Unlike the tumor volume which steadily decreases during treatment, all the PET parameters exhibit a rebound effect after the initial decline regardless of the ultimate treatment response. The rebound effect occurs between weeks 2-6. Of the PET parameters, the percent change of the RI at week 1 shows the most dramatic difference between the local control and local failure groups (Figure 3(c)).

The predictive values of tumor volume and different PET parameters for local failure are listed in Table 2. Of note, neither the tumor volume nor any of the PET parameters before RT are predictive of treatment response (all *P* = NS). Both RI and SF from the week 1 PET are very accurate in predicting local failures (AUC = 0.92, *P* = 0.03 for both). RI at week 2 and SF at week 4 are also very accurate (AUC = 0.92 and 1.00, *P* = 0.03 and 0.01, resp.). In contrast, tumor volume and SUV are only predictive for local failures at week 7, which is two weeks after completion of RT (AUC = 0.92 and 0.96, *P* = 0.03 and 0.02, resp.). To address the potential concern of noise, the PET parameters were also analyzed using the top 5% and 10% voxel activity. The results using the top 5% and 10% voxel activity were similar to the maximum voxel activity (data not shown). In Table 3, the predictive values for percent changes in PET parameters from the preradiotherapy baseline values are presented. The only PET parameter that showed a significant association with local failure during the first week of treatment was the change in RI.

**Figure 4:**
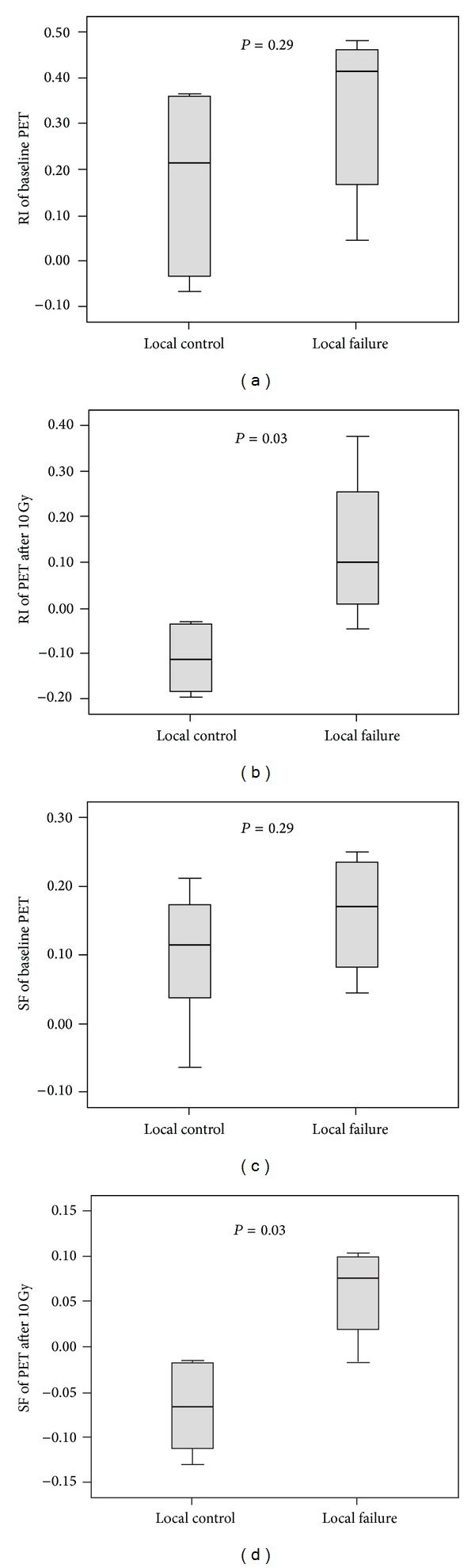
Box plots depicting the difference of PET parameters between xenografts with local control and those with local failure. (a) Retention index (RI) at baseline. (b) RI after the first week of radiotherapy (10 Gy). (c) Sensitivity factor (SF) at baseline. (d) SF after the first week of radiotherapy (10 Gy). *P* values are assessed using the Wilcoxon test.

**Figure 5:**
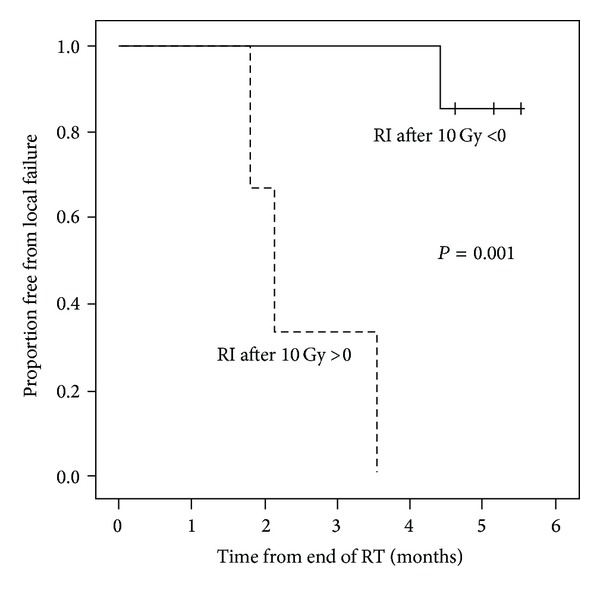
Retention Index (RI), greater than 0 after the first 10 Gy of fractionated radiotherapy, is highly predictive of local failure. Solid line represents tumors with RI < 0 after 10 Gy; dashed line represents tumors with RI > 0 after 10 Gy.

As shown in Figure 4, RI and SF are not different between the local failure and local control groups before RT, but they are significantly different after the first week of RT (*P* = 0.03 for both). RI > 0 and SF > 0 of the PET after the first week of RT are identified as the best predictors of local failure. Of note, if using the percent change from baseline instead of the absolute value, a decrease of RI less than 100% would achieve similar accuracy. Tumors with RI > 0 at week 1 have 100% local failure 4 months after RT as compared to 0% for those with RI < 0 (Figure 5). One tumor with RI < 0 at week 1 has a late tumor recurrence 4.4 months after RT (Figure 5).

## 4. Discussion

Early treatment response monitoring is a critical step in achieving the hope of personalized medicine in cancer therapy. The ideal monitoring method needs to be both accurate and performed at an early stage of the treatment course such that changes in the treatment plan can be implemented if needed. This pilot study shows that the optimal PET scanning time for early response monitoring during fractionated RT may be after 10 Gy, with 20 Gy and 40 Gy as other promising time points. It also supports RI and SF as the most promising parameters to evaluate for early treatment response during RT.

Timing is crucial for early treatment monitoring. The present study suggests that the optimal timing may be after the first 10 Gy of RT. Many tumors seem to have a rebound of FDG metabolism after the initial decline regardless of the ultimate tumor response (Figure 2). This may be due to inflammatory response [12, 14]. Therefore, the success of early treatment monitoring may rest on the ability to image before the rebound effect. Of note, RI and SF after 40 Gy (using the top 5–10% voxel activity) are highly predictive for local control (all AUC = 1.00), suggesting that imaging after the rebound phenomenon may also be informative. If two time points have similar predictive values, PET after 10 Gy would be more desirable as earlier scans would offer more flexibility to redesign a treatment course if needed, but a repeat PET scan after 40 Gy may serve as further confirmation.

Most previous clinical studies investigating the role of PET in early treatment response monitoring during RT for HNSCC have generally relied on SUV analysis at 60 min after injection [9–11]. In the present study, SUV has a relatively poor predictive value after 10–40 Gy (with AUC ranging from 0.58 to 0.75, all *P* = NS, Table 2) as compared to RI or SF. Hentschel et al. performed three serial PET scans on 37 HNSCC patients during their fractionated RT: after 10–20, 30–40, and 50–60 Gy. They found that changes of SUV ≥ 50% after 10–20 Gy (but not 30–40 Gy or 50–60 Gy) were prognostic on survival and locoregional control, which would appear to support our finding that earlier scan after 10 Gy may be more informative. However, the authors did not report the predictive accuracy of their test parameter [11]. In contrast, Castaldi et al. evaluated the SUV changes of 30 HNSCC patients after 2 weeks of RT but failed to demonstrate any significant correlation with clinical outcomes [9]. Ceulemans et al. performed a visual analysis of PET on 40 HNSCC patients after 47 Gy. They found that complete metabolic response of the PET had relatively low sensitivity and a low positive predictive value for locoregional control (29% and 31%, resp.) but they did not assess for RI or SF [10]. Taken together, these studies have shown that SUV may not be ideal for early response monitoring and future early PET studies should include analysis of RI or SF. In our analysis of changes in PET parameters during treatment, the only significance was observed in the change in RI after one week (10 Gy) of treatment (Table 3).

The findings of this study appear to complement some of the earlier results of our previous experiments correlating PET changes with histology after a single dose of RT, which showed that RI and SF correlated well with radiation-induced cellular changes [12]. Since RI and SF are mathematically related [17], it is reassuring that both provided similar results. RI is easier to determine and does not require dynamic data. If future studies would confirm equivalence between the two methods, then RI may be the more logical choice for routine clinical practice. However, SF is not restricted by fixed time points and may accommodate differences between tumors for optimal early and delayed imaging.

The biological mechanism behind the predictive value of RI and SF is unclear and cannot be addressed by the design of this study. Both RI and SF reflect on the changes of tissue tracer activity over a time period, which likely represent a combination of different types of biological processes, such as blood perfusion, hypoxia, or tumor metabolism. In classical radiobiology experiments, clonogen survival after 2 Gy* in vitro* is predictive for intrinsic cellular radiosensitivity [19], although it has not been shown to consistently predict* in vivo* tumor control [20, 21]. Tumor microenvironment and vasculature may be important to tumor response in addition to cellular radiosensitivity [22, 23]. By assessing the changes of tracer localization over a period of time with dynamic imaging, the RI or SF may capture information regarding both the intrinsic tumor radiosensitivity and vascular/microenvironment changes within the tumor. Detailed correlative studies between the PET changes and biological changes during early time points of fractionated RT will be important to understand the underlying mechanism.

Despite a strong correlation between Ki and early radiation necrosis in our previous study [12], Ki during fractionated RT in this analysis is not predictive of local control. This seems to suggest that early radiation-induced cellular change in histology is more correlative to tumor response than early radiation necrosis. There has been at least one clinical study that demonstrated the metabolic rate from FDG-PET (which is related to Ki) after approximately 24 Gy to be superior to SUV in predicting for local control of HNSCC [8]. However, that study did not analyze RI or SF. In the present study, Ki after 20 Gy has a reasonable predictive accuracy of 79%, but it is not significant, perhaps due to the small sample size. Therefore, the findings of this translational study suggest that RI or SF may be more useful than Ki (and SUV) for early treatment response during fractionated RT.

The major limitation of this study is its small sample size. Due to the extensive imaging requirements and time-consuming irradiation schedule, this pilot study was limited to 10 mice. The tumor model was also restricted to one cell line to limit the potential confounding factors. However, this pilot study was designed to determine the optimal time points and PET parameters for future studies so as to limit scanning and to improve study feasibility. Its unique design and statistically significant results should have important implications for future study design. Of course, the finding of this study should be confirmed with larger studies. Furthermore, since this is a translational study using a xenograft model in immunocompromised mice, it cannot fully capture the complex changes of tumor metabolism of HNSCC in humans. The hypothesis generated from such translational studies would certainly need to be validated in well-designed clinical studies.

## Figures and Tables

**Table 1:** Pretreatment tumor volumes and PET parameters (calculated based on the maximum voxel activity).

Characteristics	Local control median (range)	Local failure median (range)	*P* ^†^
Number	6	4	—
Volume*	656 (479–1212)	704 (530–1016)	NS
SUV_max⁡_*	1.47 (1.14–2.05)	1.51 (1.31–2.02)	NS
RI_max⁡_*	0.22 (−0.07–0.36)	0.36 (0.05–0.48)	NS
SF_max⁡_*	0.12 (−0.06–0.21)	0.17 (0.05–0.25)	NS
Ki_max⁡_*	0.014 (0.011–0.023)	0.018 (0.014–0.023)	NS

*Volume: tumor volume as determined from the CT scan, SUV_max_: standard uptake value determined from the maximum voxel activity, RI_max_: retention index determined from the maximum voxel activity, SF_max_: sensitivity factor determined from the maximum voxel activity, Ki_max_: kinetic index determined from the maximum voxel activity.

^†^ *P*: assessed using the Wilcoxon test, NS: not significant.

**Table 2:** Accuracy of PET parameters (calculated using the maximum voxel activity) at different time points during or after fractionated radiation therapy (RT) in predicting local failure.

Week*	Volume^†^		SUV_max⁡_^†^		RI_max⁡_^†^		SF_max⁡_^†^		Ki_max⁡_^†^	
	AUC^‡^	*P* ^‡^	AUC^‡^	*P* ^‡^	AUC^‡^	*P* ^‡^	AUC^‡^	*P* ^‡^	AUC^‡^	*P* ^‡^
0	0.54	NS	0.58	NS	0.71	NS	0.71	NS	0.63	NS
1	0.63	NS	0.58	NS	**0.92**	**0.03**	**0.92**	**0.03**	0.58	NS
2	0.71	NS	0.75	NS	**0.92**	**0.03**	0.83	0.09	0.79	NS
3	0.71	NS	0.71	NS	0.67	NS	0.67	NS	0.58	NS
4	0.79	NS	0.58	NS	0.75	NS	**1.00**	**0.01**	0.38	NS
5	0.88	0.06	0.79	NS	0.63	NS	0.79	NS	0.79	NS
6	0.83	0.09	0.75	NS	0.75	NS	0.71	NS	0.71	NS
7	**0.92**	**0.03**	**0.96**	**0.02**	0.50	NS	0.67	NS	0.83	0.09

*Week: time from the start of radiotherapy (which lasted 5 weeks).

^†^As denoted in Table 1.

^‡^AUC: area under the receive operating characteristic (ROC) curve. An AUC of 1 denotes perfect predictive accuracy, an AUC of 0.5 denotes complete lack of predictive accuracy, and an AUC of less than 0.5 denotes that the prediction is opposite from the initial hypothesis.* P*: assessed using the Wilcoxon test, NS: not significant. Significant values (*P* < 0.05) are displayed in bold.

**Table 3:** Accuracy of the percent changes of PET parameters from the preradiotherapy baseline in predicting local failure.

Week*	Volume^†^		SUV_max⁡_^†^		RI_max⁡_^†^		SF_max⁡_^†^		Ki_max⁡_^†^	
	AUC^‡^	*P* ^‡^	AUC^‡^	*P* ^‡^	AUC^‡^	*P* ^‡^	AUC^‡^	*P* ^‡^	AUC^‡^	*P* ^‡^
1	0.79	NS	0.50	NS	**0.96**	**0.02**	0.88	0.06	0.46	NS
2	0.83	0.09	0.67	NS	0.75	NS	0.75	NS	0.79	0.14
3	0.79	NS	0.54	NS	0.75	NS	0.63	NS	0.46	NS
4	0.88	0.06	0.54	NS	0.71	NS	0.83	0.09	0.29	NS
5	0.88	0.06	0.67	NS	0.58	NS	0.67	NS	0.67	NS
6	0.83	0.09	0.75	NS	0.71	NS	0.54	NS	0.67	NS
7	0.88	0.06	0.75	NS	0.33	NS	0.54	NS	0.63	NS

*Week: time from the start of radiotherapy (which lasted 5 weeks).

^†^As denoted in Table 1 but represented as relative percent changes from the preradiotherapy baseline.

^‡^As denoted in Table 2. Significant values (*P* < 0.05) are displayed in bold.

## References

[1] Ferlay J, Shin H-R, Bray F, Forman D, Mathers C, Parkin DM (2010). Estimates of worldwide burden of cancer in 2008: GLOBOCAN 2008. *International Journal of Cancer*.

[2] Corvò R (2007). Evidence-based radiation oncology in head and neck squamous cell carcinoma. *Radiotherapy and Oncology*.

[3] Lee N, Mechalakos J, Puri DR, Hunt M (2007). Choosing an intensity-modulated radiation therapy technique in the treatment of head-and-neck cancer. *International Journal of Radiation Oncology*Biology*Physics*.

[4] Bonner JA, Harari PM, Giralt J (2010). Radiotherapy plus cetuximab for locoregionally advanced head and neck cancer: 5-year survival data from a phase 3 randomised trial, and relation between cetuximab-induced rash and survival. *The Lancet Oncology*.

[5] Juweid ME, Cheson BD (2006). Positron-emission tomography and assessment of cancer therapy. *The New England Journal of Medicine*.

[6] Hautzel H, Müller-Gärtner H-W (1997). Early changes in fluorine-18-FDG uptake during radiotherapy. *The Journal of Nuclear Medicine*.

[7] Hunter GJ, Hamberg LM, Choi N, Jain RK, McCloud T, Fischman AJ (1998). Dynamic T1-weighted magnetic resonance imaging and positron emission tomography in patients with lung cancer: correlating vascular physiology with glucose metabolism. *Clinical Cancer Research*.

[8] Brun E, Kjellén E, Tennvall J (2002). FDG pet studies during treatment: prediction of therapy outcome in head and neck squamous cell carcinoma. *Head and Neck*.

[9] Castaldi P, Rufini V, Bussu F (2012). Can “early” and “late”^18^F-FDG PET-CT be used as prognostic factors for the clinical outcome of patients with locally advanced head and neck cancer treated with radio-chemotherapy?. *Radiotherapy and Oncology*.

[10] Ceulemans G, Voordeckers M, Farrag A, Verdries D, Storme G, Everaert H (2011). Can 18-FDG-PET during radiotherapy replace post-therapy scanning for detection/demonstration of tumor response in head-and-neck cancer?. *International Journal of Radiation Oncology*Biology*Physics*.

[11] Hentschel M, Appold S, Schreiber A (2011). Early FDG PET at 10 or 20 Gy under chemoradiotherapy is prognostic for locoregional control and overall survival in patients with head and neck cancer. *European Journal of Nuclear Medicine and Molecular Imaging*.

[12] Huang J, Chunta JL, Amin M (2012). Detailed characterization of the early response of head-neck cancer xenografts to irradiation using ^18^F-FDG-PET imaging. *International Journal of Radiation Oncology*Biology*Physics*.

[13] Yaromina A, Krause M, Thames H (2007). Pre-treatment number of clonogenic cells and their radiosensitivity are major determinants of local tumour control after fractionated irradiation. *Radiotherapy and Oncology*.

[14] Higashi K, Clavo AC, Wahl RL (1993). In vitro assessment of 2-fluoro-2-deoxy-D-glucose, L-methionine and thymidine as agents to monitor the early response of a human adenocarcinoma cell line to radiotherapy. *The Journal of Nuclear Medicine*.

[15] Burt BM, Humm JL, Kooby DA (2001). Using positron emission tomography with [^18^F]FDG to predict tumor behavior in experimental colorectal cancer. *Neoplasia*.

[16] Higashi T, Saga T, Nakamoto Y (2002). Relationship between retention index in dual-phase ^18^F-FDG PET, and hexokinase-II and glucose transporter-1 expression in pancreatic cancer. *The Journal of Nuclear Medicine*.

[17] Wong C-YO, Noujaim D, Fu HF (2009). Time sensitivity: a parameter reflecting tumor metabolic kinetics by variable dual-time f-18 FDG PET imaging. *Molecular Imaging and Biology*.

[18] Hoekstra CJ, Hoekstra OS, Lammertsma AA (1999). On the use of image-derived input functions in oncological fluorine-18 fluorodeoxyglucose positron emission tomography studies. *European Journal of Nuclear Medicine*.

[19] Carmichael J, Degraff WG, Gamson J (1989). Radiation sensitivity of human lung cancer cell lines. *European Journal of Cancer and Clinical Oncology*.

[20] Björk-Eriksson T, West C, Karlsson E, Mercke C (2000). Tumor radiosensitivity (SF_2_) is a prognostic factor for local control in head and neck cancers. *International Journal of Radiation Oncology*Biology*Physics*.

[21] Stausbøl-Grøn B, Overgaard J (1999). Relationship between tumour cell in vitro radiosensitivity and clinical outcome after curative radiotherapy for squamous cell carcinoma of the head and neck. *Radiotherapy and Oncology*.

[22] Charles N, Holland EC (2010). The perivascular niche microenvironment in brain tumor progression. *Cell Cycle*.

[23] Graves EE, Maity A, Le Q-T (2010). The tumor microenvironment in non-small-cell lung cancer. *Seminars in Radiation Oncology*.

